# Incidence and determinants of hysterectomy among North Indian women: An 8-year follow-up study

**DOI:** 10.3389/fpubh.2022.1065081

**Published:** 2022-12-16

**Authors:** Sunanda Rajkumari, Vineet Chaudhary, Sapana Kasaudhan, Kallur Nava Saraswathy

**Affiliations:** Department of Anthropology, University of Delhi, Delhi, India

**Keywords:** incident hysterectomy, rural community, reproductive health, menopause, triglycerides, folate

## Abstract

**Background:**

Despite indications of a rapid increase in the number of hysterectomies performed in India, very few studies have methodically investigated the rate and determinants of the incidence of hysterectomy. The present study aims to estimate the rate of incidence of hysterectomy and identify predictors/determinants of incident hysterectomy in a cohort of North Indian women.

**Methods:**

In the present study, a cohort of 1,009 ever-married North Indian women (aged 30–75 years) was followed up after a median of 8.11 years. Those hysterectomized at the baseline (63) were excluded; and of the rest 946 participants, 702 (74.2%) could be successfully followed-up. During the baseline assessment, data about sociodemographic variables, reproductive history, menopausal status, physiological health, and selected blood biochemicals were collected. During the end-line assessment, data about sociodemographic variables, current menopausal status, and incident hysterectomy were recorded.

**Results:**

The overall rate of incidence of hysterectomy was found to be 11.59 per 1,000 women-years, in the study population. Interestingly, the incidence rates were found to be similar among pre- and post-menopausal women. Further, while late age at menarche was found to be negatively associated with incident hysterectomy, folate repletion and high triglyceride (TG) at the baseline were found to be positively associated.

**Conclusions:**

High rate of incident hysterectomy in the studied population points toward the huge burden of gynecological morbidity and the unavailability of non-invasive protocols. Such a situation warrants immediate policy intervention. Further, maintaining TG and folate within normal physiological ranges may be beneficial in gynecological ailments necessitating hysterectomy.

## 1. Introduction

Hysterectomy is a surgical procedure that involves the removal of the uterus corpus with (total hysterectomy) or without cervix (subtotal or supracervical hysterectomy) (1). It is the second most performed surgery among women after caesarean section (2). Hysterectomy is performed for various gynaecological conditions, which include uterine cancers, other non-cancerous (benign) uterine conditions such as uterine fibroids, endometriosis, prolapse, and other uterine disorders, and chronic pelvic pain (2). In rural India, hysterectomy is reported to be a widely accepted intervention for gynaecological diseases (3).

For many decades, studies and research on hysterectomy have focused on high-income countries such as the USA, European countries, Australia, and New Zealand (4–13). As per previous reports, between 20 and 40% of women in high-income countries would undergo hysterectomy by the age of 60 years (4–8), of which most surgeries would be performed between the age of 44 and 54 years (14–18). However, studies from the last two decades have reported a decline in the prevalence and incidence of hysterectomy in high-income countries (19, 20). Meanwhile, a surge in the incidence of hysterectomy has been reported in low and middle-income countries, including India (21–24).

In a diverse country like India, the prevalence of hysterectomy varies between 1.7 to 9.8% within different regions of the country (25–30). Variations in hysterectomy rates have been associated with socio-economic, lifestyle, reproductive, and cardiometabolic variables (31). Due to a sudden increase in the prevalence, hysterectomy has emerged as an important women's health issue in India (21, 32). The claim is further strengthened by the findings of a recent study that reported an incidence rate of 20.7 per 1,000 women-years (95% CI: 14.0 to 30.8) from a cohort of low-income women of Gujarat, India (24).

Earlier believed to be a safe procedure, a growing body of literature has associated hysterectomy with several short- and long-term adverse health conditions (33–35). Hysterectomized women have potential surgical complications, an increased risk for urinary tract infections, urinary incontinence, sexual dysfunction, depression, increased fatigue, osteoporosis, coronary heart diseases, earlier onset of menopause, and anatomical complications (33–37).

Taking into consideration the growing prevalence rate of hysterectomy in developing countries, including India, and the growing concerns regarding long-term adverse health consequences of hysterectomy, it is pertinent to understand the rate of occurrence of new cases and the factors associated with them. However, very few studies have methodically investigated the rate and determinants of the incidence of hysterectomy in developing countries; accordingly, the present study follows up on a cohort of adult Jat women from rural Haryana, North India after 8 years to estimate the rate of incidence of hysterectomy and identify predictors/determinants of incident hysterectomy.

## 2. Materials and methods

### 2.1 Study design

The present study derives its data from a major Department of Biotechnology (DBT), Government of India funded project (baseline study) and a minor Institute of Eminence (IoE), University of Delhi funded project (follow-up). Both studies have been conducted among a cohort of Jat women from Palwal district, Haryana. Jat is a large ethnic community of North and Northwest India (as well as Pakistan) with a sizable population in Haryana, Delhi, Rajasthan, Gujarat, Punjab, and Western Uttar Pradesh (38). Being one of the major communities of Delhi (where the host institute undertaking the study is located) and neighboring regions (Haryana and Western Uttar Pradesh), the Jat community was selected for the study. Further, the Palwal district of Haryana was chosen as the study site for the following reasons: it is not only densely populated by the Jat community, being a border district, it shares close cultural ties with Western Uttar Pradesh, and hence increases the generalizability of the results.

During the baseline study, conducted between July 2012 to May 2013, a total of 1,009 ever married women (age group of 30–75 years) were recruited from 15 villages of Palwal, Hathin, and Hodal blocks of Palwal district, Haryana. Villages primarily inhabited by the Jat community were purposively selected. From the selected villages, 1,009 apparently healthy individuals (having no self-reported physical or mental illnesses) who were willing to participate were randomly recruited. Individuals with prior history of major chronic diseases like CVDs and cancers, on long-term medication, and pregnant/lactating mothers were not included in the study. Blood relatives up to the first cousin were also excluded from the study.

An important point worth mentioning is that the Jat community of Palwal district practice community endogamy but village exogamy (males marry outside their villages). Consequently, though the participants were recruited from 15 selected villages (where they were married), they were born and raised in, and hence represented a wider geographical region.

Of the 1,009 women, 562 (55.7%) were premenopausal, 384 (38.1%) were menopausal, and 63 (6.2%) were hysterectomized. During the baseline study, data pertaining to sociodemographic variables, reproductive history, menopausal status, physiological health, and selected blood biochemical variables were collected.

After the baseline study, the cohort was followed up from September 2020 to March 2021, i.e., after a median of 8.11 years. Since the study aimed to determine the incidence of hysterectomy, participants who were hysterectomized at the baseline study (*n* = 63) were excluded from the follow-up study. Of remain 946 women, 702 (74.2%) could be successfully followed up and re-recruited for the follow-up study. Remaining participants could not be followed up/re-recruited due to death of some of the participants, migration, and refusal to participate in the follow-up study (mostly due to the fear COVID-19 pandemic, though adequate protection measures were taken while conducting the fieldwork). The dropouts were at random. During the follow-up assessment, data pertaining to sociodemographic variables were recorded by using the same interview schedule as that of the baseline study. Additionally, data about the current menopausal status of the participants and incident hysterectomy were also recorded. The baseline study as well as the follow-up study was approved by the Institutional Ethical Committee of the Department of Anthropology, University of Delhi (approval no. Anth/2010/455/1 for baseline study and Anth/2021-22/06 for follow-up study). All the data were collected after obtaining informed written consent from the participants.

### 2.2 *Post-hoc* power calculation

*Post-hoc* power was calculated using the formula $Power\;=\;\;\;\;\;\varphi \;\{\frac{\sqrt{N*\frac{{\left(P_{1}-P_{0}\right)}^{2}}{\left(P_{0}*Q_{0}\right)}}\;-z_{1-\frac{\alpha }{2}}}{\sqrt{\frac{P_{1}*Q_{1}}{P_{0}*Q_{0}}}}\};$ where P_0_ = incidence of population [taken as 20.7 per 1,000 women-years(24)], P_1_ = incidence of study (taken as 11.59 par 1,000 women-years), N = sample size at follow up (*N* = 702), α = probability of type I error (0.05), z = critical value (z = 1.96), φ () = function to convert a critical Z-value to power. *Post-hoc* power of the study was found to be 80.1%.

### 2.3 Baseline data collection

In the baseline study, the following data were collected using the household survey method:

#### 2.3.1 Sociodemographic, lifestyle, and reproductive history

Data pertaining to the sociodemographic (name, age, educational status, occupation) and lifestyle (smoking, alcoholism) variables as well as reproductive profile (age at menarche, age at first conception, age at last conception, history of foetal loss, tubal ligation) were collected using a pretested and modified interview schedule.

#### 2.3.2 Somatometric measurements

Height (cm) and weight (kg) were taken on all the participants with lightweight clothing and without shoes by using an anthropometer rod and weighing machine, respectively. Body Mass Index (BMI) was calculated using the formula weight in kilogram divided by height in metre square (kg/m^2^). Individuals with BMI < 18 kg/m^2^ were classified as underweight, 18.0–22.9 kg/m^2^ as normal weight, 23.0–24.9 kg/m^2^ as overweight, and >25 kg/m^2^ as obese (39). Waist circumference (cm) was measured at the least circumference between the lower ribs and the iliac crest. Waist circumference < 80 cm was taken as normal (39). Hip circumference was measured at the buttock yielding the maximum circumference. The waist-hip ratio (WHR) was calculated using the formula waist circumference (cm) divided by the hip circumference (cm) i.e., (W/H). The circumference was measured using non-expandable steel tape. WHR < 0.80 among females was classified as normal (39). Blood pressure (BP) was measured by a mercury sphygmomanometer on each participant thrice at an interval of 5 min, and the average of the three readings was taken as final. Individuals with systolic and diastolic blood pressures < 120 and < 80 mmHg, respectively were classified in the normal category, between 120 and 129 and < 80 mmHg, respectively in the elevated category, between 130 and 139 or 80–89 mmHg, respectively in Stage 1 category, and ≥140 or ≥ 90 mmHg in, respectively Stage 2 category (40).

#### 2.3.3 Blood sample collection

In the baseline study, 5 ml of intravenous blood samples were collected after 12 h of fasting by a phlebotomist. The collected blood samples were transported to the Department of Anthropology, University of Delhi in an ice box. From each blood sample, serum and plasma were separated within 3 h of the sample collection and were stored at −80°C for further analysis.

### 2.4 Biochemical analysis and cut-offs

Lipid parameters included in the study were total cholesterol (TC), total triglycerides (TG), and high-density lipoprotein (HDL). Estimation of the lipid variables was done by spectrophotometer technique using the commercially available kits (Randox Laboratories Ltd.). The levels of low-density lipoprotein (LDL) and very low-density lipoprotein (VLDL) were computed using Friedewald-Levy-Fredrickson's formula (41). Normal values of TC, TG, HDL, LDL and VLDL were taken as 100–200 mg/dl, 50–150 mg/dl, >50 mg/dl, up to 130, and 10–0 mg/dl, respectively (42).

Estimation of homocysteine, folate, and vitamin B12 levels was done using Immulite 1,000 Analyser (Siemens Diagnostic products, USA) by chemiluminescence technique. High homocysteine was defined as >15 μmol/L, vitamin B12 deficiency as < 220 pg/ml, and folate deficiency as < 3 ng/ml (43).

### 2.5 Follow-up data collection

During the follow-up study, sociodemographic and lifestyle data were captured from each participant using a pretested and modified interview schedule. Current menopausal status, and data on whether the participants underwent a hysterectomy after the baseline but before the follow-up study were also captured.

### 2.6 Statistical analysis

Statistical analysis was performed using SPSS version 20.0, and appropriate statistical tools were applied for the analysis. Chi-square tests were used to determine the difference in the distribution of categorical variables. Guided by previous studies, some of the highly prevalent health conditions and associated factors (viz reproductive events, obesity, hypertension, dyslipidaemia, folate deficiency, vitamin B12 deficiency, and hyperhomocysteinemia) were selected as independent/predictor variables. Correlation analysis was performed to determine collinearity among predictor variables. No/weak correlation (correlation coefficient < 0.4) was observed among selected reproductive events, vitamin deficiencies, hyperhomocysteinemia, and hypertension. Some of the obesity and lipid parameters were moderately/strongly correlated with each other viz BMI-WC (*r* = 0.8), BMI-WHR (*r* = 0.4), WC-WHR (*r* = 0.7), TC-LDL (*r* = 0.9), TG-VLDL (*r* = 0.99). Nevertheless, since the study primarily focused on exploring the independent effects of predictor variables on incident hysterectomy, all the predictor variables were subjected to logistic regression. Both unadjusted and adjusted logistic regression models were computed. The goodness of fit was assessed by the Hosmer-Lemeshow test. For unadjusted models, incident hysterectomy was taken as the dependent variable and selected health conditions (one at a time) as the independent variable. To compute adjusted models, apart from selected health conditions (one at a time), studied sociodemographic factors (age, education, occupation, village type, alcohol consumption and smoking) were also loaded as the predictor variable. Logistic regression analysis was performed to determine the odds ratios. A significance level of *p* < 0.05 was used for all the statistical tests.

## 3. Results

### 3.1 General characteristics of the study participants

The distribution of baseline sociodemographic variables was seen among women with no history of hysterectomy and women with a hysterectomy at the end-line (incident hysterectomy). No significant differences in the distribution of the sociodemographic variables were observed between the two groups (Table 1).

**Table 1:** Distribution of baseline socio-demographic variables among women with no history of hysterectomy and incident hysterectomy.

**Variables**		**No Hys (*N* = 637)**	**Incident Hys (*N* = 66)**	***p-*value**
		***n* (%)**	***n* (%)**	
Age at enrolment	30–39	181 (28.68)	19 (28.79)	0.45
	40–49	220 (34.87)	20 (30.3)	
	50–59	140 (22.19)	20 (30.3)	
	≥60	90 (14.26)	7 (10.61)	
Education	Non-literate	491 (77.57)	51 (77.27)	0.96
	Literate	142 (22.43)	15 (22.73)	
Occupation	Agriculturist	404 (64.13)	38 (57.58)	0.35
	Housewife	217 (34.44)	26 (39.39)	
	Service	9 (1.43)	2 (3.03)	
Village type	Rural	339 (53.9)	31 (47.00)	0.28
	Urban	290 (46.1)	35 (53.00)	
Smoking status	No	372 (59.9)	36 (57.1)	0.67
	Yes	249 (40.1)	27 (42.9)	
Alcohol consumption	No	620 (99.7)	63 (98.4)	0.66
	Yes	2 (0.3)	1 (1.6)	
Median age at follow-up (IQR)		52.67 (46.30–61.00)	52.74(47.14–59.14)	0.78

Hys, Hysterectomy; IQR, Interquartile Range.

### 3.2 Rate of incidence of hysterectomy and self-reported reasons

The overall incidence rate of hysterectomy in the studied population was found to be 11.59 per 1,000 women-years (Table 2). Interestingly, the rates of incidence were found to be similar among pre-menopausal and menopausal women (11.46 and 11.81 per 1,000 women-years, respectively).

**Table 2:** Incidence and self-reported reason for hysterectomy in the studied population.

**Follow-up menopausal status**	**Overall** **(*N* = 702)**	**Baseline menopausal status**		***p*-value**
		**Pre-menopause** **(*N* = 441)**	**Natural menopause** **(*N* = 261)**	
	***n* (%)**	***n* (%)**	***n* (%)**	
Pre-menopause	225 (32.05)	225 (51.02)	-	
Natural menopause	411 (58.55)	175 (39.68)	236 (90.42)	
Hysterectomy	66 (9.40)	41 (9.30)	25 (9.58)	
Median years (IQR) of follow-up	8.11 (7.93–8.99)	8.11 (7.93–8.99)	8.11 (7.93–9.00)	
Incidence rate (CI) per 1,000 women-years	11.59 (9.04–14.66)	11.46 (8.34–15.4)	11.81 (7.81–17.18)	0.91
**Self-reported reason for hysterectomy**	**Overall Hys** **(*****N*** = **66)**	**Pre-menopause to Hys** **(*****N*** = **41)**	**Natural menopause to Hys** **(*****N***=**25)**	***p*** **-value**
	***n*** **(%)**	***n*** **(%)**	***n*** **(%)**	
Fibroids	34 (51.52)	20 (48.78)	14 (56.00)	0.34
Excessive bleeding	12 (18.18)	10 (24.39)	2 (8.00)	
Abdominal pain	14 (21.21)	7 (17.07)	7 (28.00)	
Leukorrhea and others	6 (9.09)	4 (9.76)	2 (8.00)	

IQR, Interquartile Range; CI, 95% Confidence Interval; Hys, Hysterectomy.

Uterine fibroids were the most frequently reported reason for hysterectomy among both pre-menopausal and menopausal groups. The second most frequently reported reason for hysterectomy among pre-menopausal women was excessive bleeding, whereas, among menopausal women, it was abdominal pain (Table 2).

### 3.3 Reproductive trajectory, cardiometabolic, and biochemical variables in incident hysterectomy

The distribution of baseline reproductive variables was seen between the groups of women with no history of hysterectomy and with incident hysterectomy (Table 3). The baseline reproductive events included age at menarche, age at first conception, age at the last conception, history of fetal death, and tubal ligation. No significant differences in the distribution of the above-mentioned reproductive events were found between the two groups of women. In odds ratio analysis, participants with higher age at menarche were found to be at a reduced risk of hysterectomy. Other reproductive variables were not found to be associated with hysterectomy.

**Table 3:** Distribution and association of reproductive events, baseline obesity variables, blood pressure status, and biochemical determinants among women with no history of hysterectomy and incident hysterectomy in the end-line assessment.

**Variables**		**No Hys** **(*N* = 637)**	**Incident Hys** **(*N* = 66)**	***p*-value**	**OR (95% CI)** **(Unadjusted model)**	***p*-value**	**OR (95% CI)** **(Adjusted model)^ζ^**	***p*-value**
		***n* (%)**	***n* (%)**					
Age at menarche (in years)	≥ 13 & < 16	317 (49.76)	41 (62.12)	0.09	Reference	-	Reference	-
	< 13	118 (18.52)	12 (18.18)		0.79 (0.40–1.55)	0.49	0.63 (0.30–1.32)	0.22
	≥16	202 (31.71)	13 (19.70)		0.50 (0.26–0.95)	0.03*	0.42 (0.21–0.84)	0.01*
Age at first conception (in years)	≤ 18	231 (36.55)	26 (39.39)	0.96	1.11 (0.66–1.87)	0.41	1.15 (0.67–1.97)	0.62
	19–30	396 (62.66)	40 (60.61)		Reference	-	Reference	-
	≥31	5 (0.79)	0 (0.00)		0.89 (0.05–16.39)	0.94	-	-
Age at last conception (in years)	< 30	433 (69.39)	51 (77.27)	0.18	Reference	-	Reference	-
	≥ 30	191 (30.61)	15 (22.73)		0.67 (0.37–1.22)	0.19	0.62 (0.33–1.16)	0.13
History of foetal death	No	453 (71.00)	47 (71.21)	0.97	Reference	-	Reference	-
	Yes	185 (29.00)	19 (28.79)		0.99 (0.57–1.73)	0.97	1.1 (0.62–1.97)	0.74
Tubal ligation	No	207 (32.45)	19 (28.79)	0.54	Reference	-	Reference	-
	Yes	431 (67.55)	47 (71.21)		1.19 (0.68–2.08)	0.55	1.24 (0.69–2.22)	0.48
Baseline BMI	Normal	277 (43.49)	34 (51.52)	0.27	Reference	-	Reference	-
	Underweight	132 (20.72)	8 (12.12)		0.49 (0.22–1.10)	0.08	0.45 (0.19–1.06)	0.07
	Overweight	107 (16.8)	9 (13.64)		0.69 (0.32–1.48)	0.33	0.76 (0.35–1.66)	0.49
	Obese	121 (21)	15 (22.73)		1.01 (0.53–1.92)	0.98	1.05 (0.54–2.03)	0.88
Baseline WC	Normal	315 (49.45)	35 (53.03)	0.57	Reference	-	Reference	-
	At risk	322 (50.55)	31 (46.97)		0.87 (0.52–1.44)	0.58	0.93 (0.55–1.57)	0.78
Baseline WHR	Normal	125 (19.62)	15 (22.73)	0.55	Reference	-	Reference	-
	At risk	512 (80.38)	51 (77.27)		0.83 (0.45–1.52)	0.55	0.93 (0.48–1.83)	0.84
Baseline BP	Normal	196 (30.77)	17 (25.76)	0.37	Reference	-	Reference	-
	Elevated	41 (6.44)	3 (4.55)		0.84 (0.24–3.01)	0.79	0.78 (0.17–3.57)	0.74
	Stage 1	213 (33.44)	28 (42.42)		1.52 (0.80–2.85)	0.20	1.54 (0.79–3.01)	0.21
	Stage 2	187 (29.36)	18 (27.27)		1.11 (0.56–2.22)	0.77	1.25 (0.61–2.6)	0.54
Baseline TC	Normal	480 (76.19)	47 (71.21)	0.37	Reference	-	Reference	-
	High	150 (23.81)	19 (28.79)		1.29 (0.74–2.27)	0.37	1.21 (0.67–2.21)	0.53
Baseline TG	Normal	504 (80.13)	46 (69.7)	0.04*	Reference	-	Reference	-
	High	125 (19.87)	20 (30.3)		1.75 (1.00–3.07)	0.05*	1.86 (1.04–3.32)	0.04*
Baseline HDL	Normal	421 (67.36)	48 (72.73)	0.37	Reference	-	Reference	-
	Low	204 (32.64)	18 (27.27)		0.77 (0.44–1.36)	0.38	0.88 (0.49–1.58)	0.66
Baseline LDL	Normal	507 (82.57)	52 (78.79)	0.44	Reference	-	Reference	-
	High	107 (17.43)	14 (21.21)		1.28 (0.68–2.39)	0.45	1.18 (0.61–2.28)	0.62
Baseline VLDL	Normal	504 (80.13)	47 (71.21)	0.09	Reference	-	Reference	-
	High	125 (19.87)	19 (28.79)		1.63 (0.92–2.88)	0.09	1.72 (0.96–3.09)	0.07
Baseline Hcy	Normal	234 (38.05)	22 (33.33)	0.45	Reference	-	Reference	-
	High	381 (61.95)	44 (66.67)		1.23 (0.72–2.10)	0.45	1.27 (0.71–2.27)	0.42
Baseline Folate	Normal	430 (69.92)	55 (83.33)	0.02*	Reference	-	Reference	-
	Low	185 (30.08)	11 (16.67)		0.46 (0.24–0.91)	0.03*	0.51 (0.26–1.01)	0.05*
Baseline Vitamin B12	Normal	294 (47.8)	27 (40.91)	0.29	Reference	-	Reference	-
	Low	321 (52.2)	39 (59.09)		1.32 (0.79–2.22)	0.29	1.38 (0.8–2.39)	0.25

*Significant at *p*-value < 0.05; Hys, Hysterectomy; OR, Odds Ratio; CI, 95% Confidence Interval; BMI, Body Mass Index; WC, Waist Circumference; BP, Blood Pressure; WHR, Waist Hip Ratio; TC, Total Cholesterol; TG, Triglyceride; HDL, High Density Lipoprotein; LDL, Low Density Lipoprotein; VLDL, Very Low Density Lipoprotein; Hcy, Homocysteine; ^ζ^regression model has been adjusted for age, education, occupation, village type, alcohol consumption and smoking.

No significant difference in the distribution of baseline cardiometabolic and biochemical variables was observed between women with no hysterectomy and incident hysterectomy except for TG and folate, where the proportion of women with high TG and normal folate was found to be significantly higher in incident hysterectomy group (Table 3).

In odds ratio analysis, while high TG at baseline was found to pose a 1.86-fold increased risk for a hysterectomy at endline assessment, low folate was found to reduce the risk of incident hysterectomy. Other cardiometabolic and biochemical variables were not found to be associated with incident hysterectomy.

## 4. Discussion

The present study was aimed at estimating the rate and the determinants of the incidence of hysterectomy in the studied population. In the present study, the rate of incidence of hysterectomy was found to be 11.59 per 1,000 women-years. Though this rate is lower than the incidence rate (of 20.7 per 1,000 women-years) reported in only one other such study from India (24), it is higher than the highest incident hysterectomy rates reported from developed countries such as the USA, Germany, Australia, and European countries (24). For instance, the incidence rate of hysterectomy (per 1,000 women-years) has been estimated to be 3.85 in the USA (44), 3.07–4.71 in Australia (45, 46), 3.6 in Germany (47), 2.25 in Finland (48), and 1.8 in Denmark (14). Further, developed countries have witnessed a decline in hysterectomy rates over the years, which is evident from the drop in the incidence rate in Australia (from 6.54 per 1,000 women-years in 2000–2001 to 4.71 per 1,000 women-years in 2013–2014) (45), Denmark (from 2.05 per 1,000 women-years in 1977–1981 to 1.73 per 1,000 women-years in 2011) (14) and USA (from 7.10 per 1000 women-years in 1980 to 6.60 per 1,000 women-years in 1987 and 5.10 per 1,000 women-years in 2005) (19, 49, 50). Such a decrease is presumably due to the availability of less invasive alternatives such as endometrial ablation devices, levonorgestrel intrauterine system, uterine arterial embolization, etc. (24, 51). While the incidence of hysterectomy has declined over the years in the developed parts of the world, it has emerged as an important women's health issue in developing countries like India (32). The high incidence rate of hysterectomy is suggestive of the use of an invasive procedure as a routine treatment of gynaecological ailments (32).

One particular finding worth highlighting is the similar rates of incidence of hysterectomy among pre and postmenopausal women (11.46 and 11.81 per 1,000 women-years, respectively), implying that both pre- and post-menopausal women might be at similar risk of hysterectomy.

In the present study, uterine fibroids were reported to be the top reason for hysterectomy among both pre-menopausal and menopausal groups (in 48.78 and 56.00% of cases, respectively). These findings are in concordance with the findings of some recent studies that have reported fibroids to be involved in 34 to 65.5% of hysterectomies in some other states of India (52–56). Studies from other countries have also reported fibroids as the main cause of hysterectomies (73% in Hong Kong, 60% in the USA, 23% in South Africa, 48% in Nigeria, and 30.4% in Pakistan) (57–61). Further, as per the study by Shekhar et al. (31), 61.3% of hysterectomies in Haryana were performed due to excessive menstrual bleeding/ pain, which is much higher than what has been found in the present study. Among several other states, like Maharashtra, Karnataka, Andhra Pradesh, and Tamil Nadu, more than 50% of women have been reported to have undergone hysterectomies because of excessive menstrual bleeding and pain (31).

Taking into consideration the high incidence rate of hysterectomy in the study population, it is important to understand the risk factors of hysterectomy. Baseline sociodemographic variables, reproductive trajectories, and cardiometabolic risk factors were investigated as the risk factors for incident hysterectomy. Of the studied sociodemographic variables and reproductive trajectories, none of the variables was found to be significantly associated with incident hysterectomy, except for late age at menarche. Late age at menarche was found to be protective for hysterectomy. This finding is in concordance with other reports (18, 62).

Regarding the mechanism behind this observation, studies have revealed that those women who attain early menarche are not just exposed to estrogen from an earlier age, they may also be exposed to a higher concentration of estrogen throughout their life than those who experience late menarche (62). Excess exposure to estrogen has been associated with several adverse gynaecological conditions including fibroids (18, 62). By implication, women experiencing late menarche might be at a lower risk of fibroids and other gynaecological conditions and, in turn, hysterectomy.

As far as differences in baseline biochemical variables of the two groups are concerned, the proportion of women having normal levels of folate or high TG at the baseline was found to be significantly higher in the incident hysterectomy group than in the no-hysterectomy group. The relationships between folate repletion or high TG and hysterectomy have not been widely investigated; however, can be of great public health importance.

The administration of folic acid supplementation is a routine antenatal care practice globally as well as in India (63, 64). Maternal folic acid supplementation is important prophylaxis for neural tube defects in fetus (64). Not undermining the importance of prophylactic folic acid supplementation, an emerging body of literature has associated its overdose with multiple health adversities including exacerbating vitamin B12 deficiency (64), inducing aberrant methylation patterns in animal studies (65), certain cancers (66), and fetal abnormalities (in mouse model studies) (67). The situation in India is particularly critical because pregnant women in India are often given a daily dose of 5 mg of folic acid as opposed to recommended 0.4 mg (63, 64). Although no study has yet explored serum folate or folic acid supplementation as risk factors for hysterectomy, the findings of the present study, where a significantly higher proportion of folate-replete women (having either normal or supranormal levels of folate) was found in the incident hysterectomy group than in the no-hysterectomy group, give some hint toward the involvement of folate (and possibly folic acid supplementation at supraphysiological doses) in factors necessitating hysterectomy. Even though further research is required to substantiate (or rule out) this proposition, looking at the advocacy in favor of universal folic acid fortification, an urgent need for scientific attention on this matter cannot be overemphasized.

Again, the relationship between high TG and the risk of hysterectomy has not been explored much. Yet, studies have reported a positive correlation between body fat and uterine fibroids (68) and also between some components of dietary fats and uterine fibroids (69, 70). The role of lipids in etiology of factors necessitating hysterectomy should further be investigated. Also, the high rate of hysterectomy in India must be investigated in light of the high prevalence of dyslipidaemia, particularly high TG in India (71).

An interesting trend revealed by protective and risk factors associated with hysterectomy deserves further elaboration. While late age at menarche (which was found to reduce the risk of hysterectomy) is an indicator of undernutrition (72), high TG and folate repletion (associated with increased risk) indicate overnutrition (71, 73). Together these observations point toward undernutrition being protective and overnutrition being a risk for gynecological ailments necessitating hysterectomy. From a public health perspective, this observation suggests that interventions against overnutrition can help in reducing the burden of gynecological ailments. From an evolutionary point of view, this observation points toward the potential role of undernutrition, which was widely prevalent during the early phases of human evolution (74), in averting several gynecological ailments and hence increasing fertility.

Overall, looking at the high rates of incidence of hysterectomy in India, it is important to recognize unnecessary hysterectomies as a public health problem, identify the reasons and associated modifiable risk factors, and hence design appropriate interventions to avert the factors necessitating hysterectomy.

One of the main strengths of the present study is the follow-up study design which enables the estimation of the incidence rate of a health condition/outcome (hysterectomy) and also helps in identifying its determinant along with possible causality. The present study has some limitations that should be mentioned. Firstly, the loss to follow-up of the study participants at the end-line assessment was 25.8%, which is relatively high. One of the main reasons for the high loss to follow-up was the refusal to participate in the end-line assessment due to the COVID-19 pandemic. The COVID-19 pandemic had instilled a fear of interaction with people from outside the villages among some villagers. Nevertheless, the loss to follow-up was within the calculated margin and at random. Another limitation of the present study is the exclusion of biochemical analyses in the follow-up assessment. Considering the risk of COVID-19 infection during the blood sample collection (required for biochemical analyses), the initial plan of blood sample collection at follow-up was not executed for the safety of the participants and researcher. The biochemical (lipid, vitamins, and homocysteine) levels of the participants at the end line would have been a valuable addition to the study. Further, the inclusion of detailed information on parity, gravidity, oral contraceptive use, hormone replacement therapy, family history of health conditions, dietary pattern, and physical activity would have made the study more holistic in nature.

## 5. Conclusion

The rate of incidence of hysterectomy was found to be 11.59 per 1,000 women-years, which is lower than the only Indian study on incident hysterectomy, but much higher than incidence rates in developed countries. Such a high incidence rate of hysterectomy highlights the huge burden of gynecological morbidity in the study population and the unavailability of non-invasive protocols in rural areas. It further suggests that hysterectomy is seen as a medically rational and socially acceptable intervention for even those gynecological ailments for which non-invasive treatment protocols are otherwise available. The lack of proper guidelines on hysterectomy leaves the scope of misuse. Looking at the rising incidence rate of hysterectomy, health policies of India should address the (misuse of) hysterectomy as an urgent matter of concern. Further, high TG and folate repletion (most likely overdose) appear to increase the risk of hysterectomy. Maintaining these two parameters within normal physiological ranges may prove to be beneficial in alleviating the burden of gynecological ailments necessitating hysterectomy.

## Data availability statement

The raw data supporting the conclusions of this article will be made available by the authors, without undue reservation.

## Ethics statement

The studies involving human participants were reviewed and approved by Institutional Ethics Committee, Department of Anthropology, University of Delhi (approval no. Anth/2010/455/1 for baseline study and Anth/2021-22/06 for follow-up study). The patients/participants provided their written informed consent to participate in this study.

## Author contributions

KS conceptualized the study and formulated the study design and methodology. SR, VC, and SK carried out the data collection, performed the lab analysis, and participated in data analysis. KS, SR, and VC participated in data interpretation. SR and VC participated in writing the original draft. KS, SR, and SK participated in the review and editing. All the authors have read and given final approval to this version of the MS to be published and have also agreed to be accountable for all aspects of the work.

## References

[1] Nesbitt-Hawes EM Maley PE Won HR Law KSK Zhang CS Lyons SD. Laparoscopic subtotal hysterectomy: evidence and techniques. J Minim Invasive Gynecol. (2013) 20:424–34. 10.1016/j.jmig.2013.01.009 23510954

[2] Hammer A Rositch AF Kahlert J Gravitt PE Blaakaer J Søgaard M. Global epidemiology of hysterectomy: possible impact on gynaecological cancer rates. Am J Obstet Gynecol. (2015) 213:23–9. 10.1016/j.ajog.2015.02.019 25724402

[3] Verma D Verma ML. Trends of hysterectomy in the rural tertiary level teaching hospitals in Northern India. Indian J Obstet Gynecol Res. (2016) 3:212–5. 10.5958/2394-2754.2016.00048.5

[4] Rositch AF Nowak RG Gravitt PE. Increased age and race-specific incidence of cervical cancer after correction for hysterectomy prevalence in the united states from 2000 to 2009. Cancer. (2014) 120:2032. 10.1002/cncr.28548 24821088 PMC4073302

[5] Stang A Merrill RM Kuss O. Prevalence-corrected hysterectomy rates by age and indication in Germany 2005–2006. Arch Gynecol Obstet. (2012) 286:1193–200. 10.1007/s00404-012-2415-2/figures/4 22718096

[6] Redburn JC Murphy MFG. Hysterectomy prevalence and adjusted cervical and uterine cancer rates in England and Wales. BJOG. (2001) 108:388–95. 10.1111/j.1471-0528.2001.00098.x 11305546

[7] Ong S Codd MB Coughlan M O'Herlihy C. Prevalence of hysterectomy in Ireland. Int J Gynaecol Obstet. (2000) 69:243–7. 10.1016/S0020-7292(00)00195-8 10854866

[8] Spilsbury K Semmens JB Hammond I Bolck A. Persistent high rates of hysterectomy in Western Australia: a population-based study of 83 000 procedures over 23 years. BJOG. (2006) 113:804–9. 10.1111/j.1471-0528.2006.00962.x 16827764

[9] Cosson M Lambaudie E Boukerrou M Querleu D Crépin G. Vaginal, laparoscopic, or abdominal hysterectomies for benign disorders: immediate and early postoperative complications. Eur J Obstet Gynecol Reprod Biol. (2001) 98:231–6. 10.1016/S0301-2115(01)00341-4 11574137

[10] Wilcox LS Koonin LM Pokras R Strauss LT Xia Z Peterson HB. Hysterectomy in the United States, 1988-1990. Obstet Gynecol. (1994) 83:549–55. 10.1097/00006250-199404000-00011 8134065

[11] McPherson K Strong PM Epstein A Jones L. Regional variations in the use of common surgical procedures: within and between England and Wales, Canada and the United States of America. Soc Sci Med Part A Psychol Med Sociol. (1981) 15:273–88. 10.1016/0271-7123(81)90011-0 6973209

[12] Van Keep PA Wildemeersch D Lehert P. Hysterectomy in six European countries. Maturitas. (1983) 5:69–75. 10.1016/0378-5122(83)90001-4 6633270

[13] Dharmalingam A Pool I Dickson J. Biosocial determinants of hysterectomy in New Zealand. Am J Public Health. (2000) 90:1455. 10.2105/ajph.90.9.1455 10983207 PMC1447627

[14] Lykke R Blaakær J Ottesen B Gimbel H. Hysterectomy in Denmark 1977–2011: changes in rate, indications, and hospitalization. Eur J Obstet Gynecol Reprod Biol. (2013) 171:333–8. 10.1016/j.ejogrb.2013.09.011 24094821

[15] Hill EL Graham ML Shelley JM. Hysterectomy trends in Australia – between 2000/01 and 2004/05. Aust N Z J Obstet Gynaecol. (2010) 50:153–8. 10.1111/j.1479-828x.2009.01130.x 20522072

[16] Wu JM Wechter ME Geller EJ Nguyen TV Visco AG. Hysterectomy rates in the United States, 2003. Obstet Gynecol. (2007) 110:1091–1095. 10.1097/01.aog.0000285997.38553.4b 17978124

[17] Wright JD Herzog TJ Tsui J Ananth CV Lewin SN Lu YS. Nationwide trends in the performance of inpatient hysterectomy in the United States. Obstet Gynecol. (2013) 122:233–41. 10.1097/AOG.0b013e318299a6cf 23969789 PMC3913114

[18] Wilson LF Mishra GD. Age at menarche, level of education, parity and the risk of hysterectomy: a systematic review and meta-analyses of population-based observational studies. PLoS ONE. (2016) 11:e0151398. 10.1371/journal.pone.0151398 26963512 PMC4786144

[19] Whiteman MK Hillis SD Jamieson DJ Morrow B Podgornik MN Brett KM. Inpatient hysterectomy surveillance in the United States, 2000–2004. Am J Obstet Gynecol. (2008) 198:34–e1. 10.1016/j.ajog.2007.05.039 17981254

[20] Stankiewicz A Pogany L Popadiuk C. Prevalence of self-reported hysterectomy among Canadian women, 2000/2001–2008. Chronic Dis Inj Canada. (2014) 34:5. 10.24095/hpcdp.34.1.05 24618379

[21] Meher T Sahoo H. Regional pattern of hysterectomy among women in India: evidence from a recent large scale survey. Women Health. (2020) 60:585–600. 10.1080/03630242.2019.1687634 31718517

[22] Towghi F. Cutting inoperable bodies: particularizing rural sociality to normalize hysterectomies in Balochistan, Pakistan. Med Anthropol. (2012) 31:229–48. 10.1080/01459740.2011.623488 22540316

[23] Desai S. Pragmatic prevention, permanent solution: Women's experiences with hysterectomy in rural India. Soc Sci Med. (2016) 151:11–8. 10.1016/j.socscimed.2015.12.046 26773294

[24] Desai S Campbell OMR Sinha T Mahal A Cousens S. Incidence and determinants of hysterectomy in a low-income setting in Gujarat, India. Health Policy Plann. (2017) 32:68–78. 10.1093/heapol/czw099 27497139 PMC5886266

[25] Desai S Sinha T Mahal A. Prevalence of hysterectomy among rural and urban women with and without health insurance in Gujarat, India. Reprod Health Matters. (2011) 19:42–51. 10.1016/S0968-8080(11)37553-2 21555085

[26] Bhasin SK Roy R Agrawal S Sharma R. An epidemiological study of major surgical procedures in an urban population of East delhi. Indian J Surg. (2011) 73:131–5. 10.1007/s12262-010-0198-x 22468063 PMC3077158

[27] Singh A Arora AK. Why Hysterectomy rate are lower in India. Indian J Community Med. (2008) 33:196. 10.4103/0970-0218.42065 19876485 PMC2763672

[28] Patel V Tanksale V Sahasrabhojanee M Gupte S Nevrekar P. The burden and determinants of dysmenorrhoea: a population-based survey of 2262 women in Goa, India. BJOG. (2006) 113:453–63. 10.1111/j.1471-0528.2006.00874.x 16489934

[29] Kaur S Walia I Singh A. How menopause affects the lives of women in suburban Chandigarh, India. Climacteric. (2004) 7:175–80. 10.1080/13697130410001713779 15497906

[30] Prusty RK Choithani C Gupta SD. Predictors of hysterectomy among married women 15–49 years in India. Reprod Health. (2018) 15:1–1. 10.1186/s12978-017-0445-8 29304867 PMC5756367

[31] Shekhar C Paswan B Singh A. Prevalence, sociodemographic determinants and self-reported reasons for hysterectomy in India. Reprod Health. (2019) 16:1–16. 10.1186/s12978-019-0780-z 31375139 PMC6679457

[32] Singh A Govil D. Hysterectomy in India: Spatial and multilevel analysis. Women's Health. (2021) 17:68. 10.1177/17455065211017068 34096404 PMC8188977

[33] Madueke-Laveaux OS Elsharoud A Al-Hendy A. What we know about the long-term risks of hysterectomy for benign indication—a systematic review. J Clin Med. (2021) 10:5335. 10.3390/jcm10225335 34830617 PMC8622061

[34] Laughlin-Tommaso SK Satish A Khan Z Smith CY Rocca WA Stewart EA. Long-term risk of de novo mental health conditions after hysterectomy with ovarian conservation: a cohort study. Menopause. (2020) 27:33–42. 10.1097/gme.0000000000001415 31479034 PMC7089568

[35] Clarke-Pearson DL Geller EJ. Complications of hysterectomy. Obstet Gynecol. (2013) 121:654–73. 10.1097/aog.0b013e3182841594 23635631

[36] Farquhar CM Sadler L Harvey SA Stewart AW. The association of hysterectomy and menopause: a prospective cohort study. BJOG: Int J Gynaecol Obstet. (2005) 112:956–62. 10.1111/j.1471-0528.2005.00696.x 15957999

[37] Link CL Pulliam SJ McKinlay JB. Hysterectomies and urologic symptoms: results from the Boston area community health (BACH) survey. Female Pelvic Med Reconstr Surg. (2010) 16:37–47. 10.1097/spv.0b013e3181cb9931 21423814 PMC3060564

[38] Kadyan A. Know The Jat. 1st edition. Delhi: Bluerose Publishers (2020).

[39] Misra A Chowbey P Makkar B Vikram N Wasir J Chadha D. Consensus statement for diagnosis of obesity, abdominal obesity and the metabolic syndrome for Asian Indians and recommendations for physical activity, medical, and surgical management. J Assoc Physicians India. (2009) 57:163–70. 19582986

[40] Whelton PK Carey RM Aronow WS Casey DE Collins KJ Himmelfarb CD. 2017 ACC/AHA/AAPA/ABC/ACPM/AGS/APhA/ASH/ASPC/NMA/PCNA Guideline for the Prevention, detection, evaluation, and management of high blood pressure in adults: executive summary: a report of the American college of cardiology/American heart association task force on clinical practice guidelines. Hypertension. (2018) 71:1269–324. 10.1161/hyp.0000000000000066 29133354

[41] Friedewald WT Levy RI Fredrickson DS. Estimation of the concentration of low-density lipoprotein cholesterol in plasma, without use of the preparative ultracentrifuge. Clin Chem. (1972) 18:499–502. 10.1093/clinchem/18.6.499 4337382

[42] National Cholesterol Education Program. ATP III At-A-Glance: Quick Desk Reference. (2001). Available online at: https://www.nhlbi.nih.gov/resources/atp-iii-glance-quick-desk-reference (accessed October 8, 2022).

[43] Sukla KK Raman R. Association of MTHFR and RFC1 gene polymorphism with hyperhomocysteinemia and its modulation by vitamin B12 and folic acid in an Indian population. Eur J Clin Nutr. (2012) 66:111–8. 10.1038/ejcn.2011.152 21878957

[44] Doll KM Dusetzina SB Robinson W. Trends in inpatient and outpatient hysterectomy and oophorectomy rates among commercially insured women in the United States, 2000–2014. JAMA Surg. (2016) 151:876–7. 10.1001/jamasurg.2016.0804 27168235 PMC5480952

[45] Wilson LF Pandeya N Mishra GD. Hysterectomy trends in Australia, 2000–2001 to 2013–2014: joinpoint regression analysis. Acta Obstet Gynecol Scand. (2017) 96:1170–9. 10.1111/aogs.13182 28627047

[46] Yusuf F Leeder S Wilson A. Recent estimates of the incidence of hysterectomy in New South Wales and trends over the past 30 years. Aust N Z J Obstet Gynaecol. (2016) 56:420–5. 10.1111/ajo.12477 27297684

[47] Stang A Merrill RM Kuss O. Nationwide rates of conversion from laparoscopic or vaginal hysterectomy to open abdominal hysterectomy in Germany. Eur J Epidemiol. (2011) 26:125–33. 10.1007/S10654-010-9543-4 21240542

[48] Hakkarainen J Nevala A Tomás E Nieminen K Malila N Pitkäniemi J. Decreasing trend and changing indications of hysterectomy in Finland. Acta obst gyn scand. (2021) 100:1722–9. 10.1111/aogs.14159 33797081

[49] Lepine LA Hillis SD Marchbanks PA Koonin LM Morrow B Kieke BA Wilcox LS. Hysterectomy surveillance—United States, 1980–1993. MMWR Surveill Summ. (1997) 1–15. Available online at: https://www.jstor.org/stable/24676449 9259214

[50] Farquhar CM Steiner CA. Hysterectomy rates in the United States 1990–1997. Obstet Gynecol. (2002) 99:229–34. 10.1016/S0029-7844(01)01723-9 11814502

[51] Nagrani R Bowen-Simpkins P Barrington JW. Can the levonorgestrel intrauterine system replace surgical treatment for the management of menorrhagia? BJOG: Int J Gynaecol Obstet. (2002) 109:345–7. 10.1016/S1470-0328(02)01274-0 11950191

[52] Uikey P Wankhede TM Tajne MP. The route of hysterectomy: a comparative study between abdominal hysterectomy (AH), non-descent vaginal hysterectomy (NDVH), and laparoscopic assisted vaginal hysterectomy (LAVH). Int J Reprod Contracept Obstet Gynecol. (2018) 7:4022–9. 10.18203/2320-1770.ijrcog20184123

[53] Radha K Devi GP Chandrasekharan PA Swathi P Radha G Keerthana. Epidemiology of hysterectomy-a cross sectional study among piligrims of tirumala. IOSR J Dent Med Sci. (2015) 14:105. 10.9790/0853-14760105

[54] Kaur M Agarwal A Das C Kundal RK Mall N Jindal S. Clinicopathological study of 100 cases of hysterectomies. J Indian Med Assoc. (2002) 3:100.

[55] Bala R Devi KP Singh CM. Trend of hysterectomy: a retrospective analysis in Regional Institute of Medical Sciences (RIMS). J Med Soc. (2015) 29:4–7. 10.4103/0972-4958.158917

[56] Pandey D Sehgal K Saxena A Hebbar S Nambiar J Bhat RG. An audit of indications, complications, and justification of hysterectomies at a teaching hospital in India. Int J Reprod Med. (2014) 3:9273. 10.1155/2014/279273 25763395 PMC4334049

[57] Butt JL Jeffery ST Van der Spuy ZM. An audit of indications and complications associated with elective hysterectomy at a public service hospital in South Africa. Int J Gynaecol Obstet. (2012) 116:112–6. 10.1016/j.ijgo.2011.09.026 22142874

[58] Broder MS Kanouse DE Mittman BS Bernstein SJ. The appropriateness of recommendations for hysterectomy. Obstet Gynecol. (2000) 95:199–205. 10.1016/S0029-7844(99)00519-0 10674580

[59] Leung PL Tsang SW Yuen PM. An audit on hysterectomy for benign diseases in public hospitals in Hong Kong. Hong Kong Med J. (2007) 13:187–193. 17548906

[60] Nisa Qamar H. Habibullah, Fatima M, Tanweer Ahmed S, Zehra M. Hysterectomies; an audit at a tertiary care hospital Professional Med J. (2011) 18:46–50.

[61] Adelusola KA Ogunniyi SO. Hysterectomies in Nigerians: histopathological analysis of cases seen in Ile-Ife. Niger Postgrad Med J. (2001) 8:37–40. 11487782

[62] Cooper R Hardy R Kuh D. Timing of menarche, childbearing and hysterectomy risk. Maturitas. (2008) 61:317. 10.1016/j.maturitas.2008.09.025 19013032 PMC3500690

[63] Behere RV Yajnik CS. Low vitamin B-12-high folate status in adolescents and pregnant women may have deleterious effects on health of the offspring. Am J Clin Nutr. (2021) 113:1057–9. 10.1093/ajcn/nqab007 33822862 PMC7611135

[64] Field MS Stover PJ. Safety of folic acid. Ann N Y Acad Sci. (2018) 1414:59–71. 10.1111/nyas.13499 29155442 PMC5849489

[65] Smith AD Kim YI Refsum H. Is folic acid good for everyone? Am J Clin Nutr. (2008) 87:517–33. 10.1093/ajcn/87.3.517 18326588

[66] Kim YI. Folate and cancer: a tale of Dr. Jekyll and Mr Hyde? Am J Clin Nutr. (2018) 107:139–42. 10.1093/ajcn/nqx076 29529163

[67] Tsang V Fry RC Niculescu MD Rager JE Saunders J Paul DS. The epigenetic effects of a high prenatal folate intake in male mouse foetuses exposed in utero to arsenic. Toxicol Appl Pharmacol. (2012) 264:439–50. 10.1016/j.taap.2012.08.022 22959928 PMC3478409

[68] Yang Y He Y Zeng Q Li S. Association of body size and body fat distribution with uterine fibroids among Chinese women. J Womens Health (Larchmt). (2014) 23:619–26. 10.1089/jwh.2013.4690 25010826

[69] Brasky TM Bethea TN Wesselink AK Wegienka GR Baird DD Wise LA. Dietary fat intake and risk of uterine leiomyomata: a prospective ultrasound study. Am J Epidemiol. (2020) 189:1538–46. 10.1093/aje/kwaa097 32556077 PMC7857646

[70] Harris HR Eliassen AH Doody DR Terry KL Missmer SA. Dietary fat intake, erythrocyte fatty acids, and risk of uterine fibroids. Fertil Steril. (2020) 114:837–47. 10.1016/j.fertnstert.2020.03.023 32680614 PMC7554115

[71] Joshi SR Anjana RM Deepa M Pradeepa R Bhansali A Dhandania VK. Prevalence of dyslipidemia in urban and rural India: the ICMR–INDIAB study. PLoS ONE. (2014) 9:e96808. 10.1371/journal.pone.0096808 24817067 PMC4016101

[72] Canelón SP Boland MR. A systematic literature review of factors affecting the timing of menarche: the potential for climate change to impact women's health. Int J Environ Res Public Health. (2020) 17:1703. 10.3390/ijerph17051703 32150950 PMC7084472

[73] McNulty H Pentieva K. “Folate bioavailability”. In:Bailey LB, editor. Folate in Health and Disease 2nd Edition. Boca Raton: CRC Press (2009). P. 25-47. 10.1201/9781420071252

[74] Neel J V. Diabetes mellitus: a “thrifty” genotype rendered detrimental by “progress”? Am J Hum Genet. (1962) 14:353–362. 13937884 PMC1932342

